# Gender and diagnostic differences in children’s preferences for social robot design. A mixed-methods study with autistic and neurotypical children

**DOI:** 10.3389/frobt.2026.1812453

**Published:** 2026-08-07

**Authors:** Gema Benedicto Rodríguez, Vanessa Zorrilla-Muñoz, Carlos G. Juan, Jose Manuel Ferrandez

**Affiliations:** 1 Polytechnic University of Cartagena, Cartagena, Murcia, Spain; 2 Bioengineering Institute, Miguel Hernández University of Elche, Elche, Spain; 3 Carlos III University of Madrid, Getafe, Spain

**Keywords:** autism, design robot, human-robot interaction, robot, social inclusion

## Abstract

**Introduction:**

Autistic children are increasingly engaging with social robots in educational and support contexts, but limited research has compared the perceptual and design preferences of autistic and neurotypical children.

**Methods:**

This mixed-methods study examined the robot design preferences of 43 children aged 3–15 years, including 21 autistic and 22 neurotypical children, and included semi-structured interviews with 11 adult stakeholders, comprising six experts and five non-experts. Quantitative analyses explored relationships between robot preference and perceptual features, including size, color, movement, voice, and anthropomorphism, while qualitative thematic and co-occurrence analyses examined perceived benefits, ethical issues, accessibility challenges, and ideal design characteristics.

**Results:**

Perceptual characteristics, particularly movement intensity and color multiplicity, influenced robot preference. Across groups, children generally preferred humanoid robots with soft voices and eye movement. Gender-related differences appeared more consistent than diagnostic differences in preferences for size and movement speed. Autistic children highlighted playful and social interaction functions and showed greater variability in preferred personality-related traits. Qualitative findings indicated strong interest in robot-supported interactions, while also raising concerns about accessibility and possible emotional dependency.

**Discussion:**

These findings suggest that inclusive social robot design should prioritize modificable sensory features, moderated anthropomorphism, predictable interaction patterns, and personalization mechanisms that account for individual, gender-related, and diagnostic differences.

## 1 Introduction

Social robotics has established itself as a promising tool for supporting social and communication skills in childhood, especially in autistic children. To understand its use with autistic and neurotypical children, it's important to clarify what the autism spectrum is, what these robots do, how effective they are, and why design and user preferences are key.

### 1.1 Autism spectrum disorder

ASD^1^ is a neurodevelopmental disorder characterized by differences in communication and social interaction styles, the development of restricted interests, repetitive behaviors (Hirota and King, 2023) and/or sensory behaviors (Lord et al., 2020). These traits can manifest early in life and persist throughout the lifespan. When diagnosed during childhood, children may develop social skills differently, sometimes requiring structured support because their interaction with the environment is limited (Cabibihan et al., 2013). The heterogeneity of sensory processing and social perception in ASD has implications for how technological agents are perceived and accepted.

In countries with high income, ASD affects 2.3% of children and 2.2% of adults in the general population (Hirota and King, 2023). Prevalence has risen due to changes in diagnostic criteria, improved screening, and greater awareness (Lord et al., 2020). In this regard, and with the aim of not only improving detection tools but also addressing the challenges and differences that may arise in a person throughout their life, therapeutic interventions are increasingly moving beyond conventional approaches (Hirota and King, 2023). However, there remains a need to identify which treatments are most effective in the long term to promote greater independence and quality of life for autistic persons (Lord et al., 2020), and little is known about how children themselves perceive robot design features.

Moreover, technology-based support approaches are increasingly being explored, including the use of social robots. This therapeutic approach offers an innovative and promising solution because social robots have the ability to capture attention and motivate autistic children through predictable interactions tailored to their needs (Rakhymbayeva et al., 2021).

### 1.2 Use of robots for autistic children

Both autistic and neurotypical children tend to perceive social robots primarily as toys. However, autistic children also categorize them as machines and show differences in their perception and conceptualization of robots: they prefer simplified designs and exaggerated facial features, which also seems to aid their interaction with robots by providing clearer social cues (Peca et al., 2014). In fact, predictable and structured interactions with robots help reduce discomfort and support the learning of social skills (Schadenberg, 2021). In this regard, the evidence is mixed, with some studies showing improvements (e.g., eye contact, verbal initiation) while others have found limited differences compared to human-led therapy (Castillo Bautista and Sánchez-Suricalday, 2023; Chung, 2019). Moreover, existing platforms vary widely in anthropomorphism, size, and sensory features, yet systematic evaluation of how these characteristics align with user preferences remains limited (Santos López, 2023; Feil-Seifer and Mataric, 2011; Wood et al., 2013; Tamura et al., 2004). Despite this, and although there are findings on the preferences for social robots among autistic children, comparative research between the preferences of neurotypical and autistic children remains limited, highlighting the need for more inclusive studies to inform the development of robots adapted to pediatric populations (Peca et al., 2014; Mouelhi et al., 2020).

After analyzing the different types of robots that can be effective in various aspects of children’s development, it has been concluded that the physical aspects of robots are diverse, yet those that have been beneficial in therapeutic environments share a common characteristic: the robot’s physical appearance and robustness during interaction directly impact its attractiveness and effectiveness (Castillo Bautista and Sánchez-Suricalday, 2023).

### 1.3 Preferences and effectiveness of robots in the use

As social robots become increasingly human-like, they approach what is known as the ‘uncanny valley’, a point where the robot’s appearance becomes disturbing and as Yousif (2021) described: the concept also highlights how human-like curves and roundness do not induce eerie sensations (much like healthy humans in motion, yielding higher affinity), whereas death renders the body immobile, pale, and cold, evoking lower affinity and paralleling the eerie immobility sometimes perceived in robots. This phenomenon contrasts with industrial robots, which perform human-like functions but lack human appearance (generating little affinity), and toy robots, where human-like features (face, arms, torso) take precedence over functionality. Similarly, prosthetic hands and teeth may appear highly human-like at first glance but provoke unease upon touch, reducing affinity.

Accordingly, in social robots, movement parameters (speed, acceleration, deceleration) significantly modulate the uncanny valley effect: observers feel greater affinity for robots exhibiting familiar, human-like motion, while unexpected or mismatched movements intensify discomfort. Moreover, mismatches between highly human-like appearance and robotic movement strongly impact perception (Mori et al., 2012; Fong et al., 2003), especially in vulnerable groups (Tamura et al., 2004). However, subsequent empirical tests have shown that the predicted deepening of the valley under motion conditions is not consistently observed (Piwek et al., 2014). In this sense, designs that incorporate biological principles (such as fluid movements and rounded shapes inspired by human biology) can help generate greater affinity and avoid the uncanny valley, making them especially relevant for robot design in contexts involving autistic children (Breazeal, 2003). These considerations align with empirical preferences reported in the literature, where avoiding excessive anthropomorphism and prioritizing predictable, biologically plausible interactions emerges as a key factor.

Previous studies have compiled common preferences reported by autistic children regarding categories and requirements for social robots, integrating principles from Bandura’s social learning theory (Bandura, 1977) and the perceived theory of social robots’ interactions (Robins and Dautenhahn, 2014) and robot therapy (CORDIS, 2025), as shown in Table 1. These preference models are theoretical, though, and are rarely verified by comparing neurotypical and autistic populations directly.

**TABLE 1:** Preferences according to category and requirements of social robots based on Robins and Dautenhahn (2014), CORDIS (2025), and Belpaeme et al. (2018).

Category	Requirements
Physical design	Different parts of the robot’s body can have varying colors and brightness but should not be too bright or sharp-edged.
	Preferred features include shapes, lights, and rotating mechanical parts.
	Movements should be slow and smooth, avoiding abrupt motions.
	Facial expressions should be simple, without detailed eyebrows or eyelashes.
	The robot should not resemble a human too closely to avoid intimidation or loss of interest, but it should not be too mechanical either.
	The eyes and direction of gaze should be modular, as some children are attracted to them while others may dislike them.
	The robot’s height should be similar to that of the child to facilitate interaction and enhance the play experience.
	The presence of control buttons enhances the child’s ability to make choices.
	Robustness is crucial to withstand falls and physical interaction.
	The robot should offer tactile exploration and have the ability to speak.
Functional aspects	Take turns to encourage reciprocal conversation and prevent one-sided, repetitive communication.
	Imitate actions and verbal behaviors to help children acquire new skills.
	Provide sensory rewards and stimuli to encourage engagement.
	Use games for therapy, as they are essential for cognitive and social development, preventing isolated play situations.
	Personalize interactions based on the child’s needs and interests, gradually increasing task complexity.
	Focus on individual therapy to maintain attention and engagement.
	Create safe environments to allow for free play.
	Act as a social mediator or behavior influencer.

Most studies on social robots for autistic children have focused on therapeutic outcomes, such as joint attention (David et al., 2018; So et al., 2023; Aguirre et al., 2024) or emotion recognition (Pinto-Bernal et al., 2023). However, design preferences from the children’s own perspective remain largely unexplored (Sanromà-Giménez et al., 2021). Furthermore, research addressing user preferences rarely considers a neurodiversity or inclusive design framework, seeking to respect neurological differences and promote equitable access that affirms and respects neurological diversity. On the other hand, gender differences in the perception of and preference for social robots are also understudied, and some findings suggest that autistic girls may exhibit different behaviors and responses to these robots in comparison to boys (Wood-Downie et al., 2021), and that gender stereotypes influence how characteristics are attributed to robots (Kislev, 2023). Despite this, there is still an urgent need for approaches that consider both neurological diversity and gender differences to create accessible and meaningful technologies for all autistic children (Sanromà-Giménez et al., 2021; Saleh et al., 2021; Kumazaki et al., 2020). Therefore, the present study focuses on these gaps by explicitly comparing preferences and gender and incorporating stakeholder perspectives on the design of inclusive and neurodiverse robots.

### 1.4 Why robots?

Despite these positive outcomes, some children may react negatively to robots, particularly to noises or unexpected movements. This issue has been addressed in some cases by reducing noise and ensuring smooth, continuous movements, resulting in non-anthropomorphic mobile robots with similar interactive capabilities to those equipped with torsos (Feil-Seifer and Mataric, 2011).

Interestingly, negative reactions are not limited to children and adolescents; educators viewing images of social robots (e.g., NAO, Kaspar, Milo) generally showed positive attitudes toward classroom use. They believed robots could increase children’s willingness to learn, help them generalize acquired skills due to their interactive nature, and provide predictable behaviors that support attention maintenance, joint attention, and other social behaviors in autistic children. Nevertheless, challenges persist in classroom integration, including cost-benefit considerations, limited evidence from studies with older users or larger samples, and concerns about long-term socialization skills and full human-like social development (Belpaeme et al., 2018).

In summary, the goal of these studies has been to achieve a moderate degree of human resemblance and foster a sense of affinity, which can improve future human-robot interactions in both clinical and experimental settings.

Given the complexities and variability in how autistic children interact with their environment, it is necessary to contextualize specific preferences and responses to social robots prior to design. The aim of this study is not only to explore how these robots can be integrated into therapeutic practices but also to analyze the design preferences of social robots for both neurotypical and autistic children. By examining these preferences, this research seeks to identify key design elements that can enhance therapeutic outcomes, thereby bridging the gap between technological innovation and personalized therapy. This research investigates the nuances in how different groups perceive and interact with robots, aiming to inform the design of more inclusive, adaptable, and user-centred social robots.

There is still little data comparing the perceptual and design preferences of autistic children to those with neurotypical behavior, despite the growing use of social robots in ASD contexts. To advance inclusive and user-centered robot design, it is necessary to comprehend these distinctions.

### 1.5 Objectives of the study

The objective of this study is to analyze preferences for social robots’ design for therapeutic purposes in neurotypical children and autistic children, seeking differences between these groups while considering insights from both experts and non-experts.

Specifically, the study addresses the following research questions:

- What features of robots are preferred by autistic children compared to neurotypical children?
- Are there differences in robot design preferences based on gender within each group (ASD vs. NT)?
- Do the perceptions of experts and non-experts coincide or differ in relation to children’s preferences?

What co-occurrence patterns across responses from both groups help identify useful design guidelines for social robots intended for use with autistic children? These research questions are framed within an inclusive design and neurodiversity perspective, which prioritizes user-centered, adaptable technologies that accommodate the full spectrum of perceptual and gender-related differences rather than assuming a one-size-fits-all solution.

## 2 Methods

This study employed a mixed-methods design to explore children’s preferences for social robot design features and stakeholders’ perceptions of their therapeutic use in ASD. The approach combined quantitative data from children’s questionnaires (assessing perceptual and design preferences) with qualitative data from semi-structured interviews with experts and non-experts (examining experiences, benefits, ethics, challenges, and ideal designs). This integration allowed for a comprehensive understanding of both child-centered preferences and broader contextual insights.

The Methods section is organized as follows: Section 2.1 describes the recruitment and characteristics of children and adult interviewees; Section 2.2 details the questionnaire and interview protocol; Section 2.3 outlines the sequence of data collection and ethical considerations; and Section 2.4 explains the quantitative and qualitative processing, including thematic coding and co-occurrence analysis using ATLAS.ti.

### 2.1 Participants

This study involved a total of 43 children, divided into two groups: 21 children diagnosed with ASD (18 boys and 3 girls), and 22 neurotypical children (13 boys and 9 girls, control group) without any ASD diagnosis.

The ages of the neurotypical children ranged from 6 to 15 years, while the ages of the autistic children ranged from 3 to 14 years. The inclusion criterion for the ASD group was a formal clinical diagnosis of ASD, regardless of the severity of the condition. In contrast, the neurotypical group required the absence of any psychiatric or neurological diagnosis. Both groups had an upper age limit of 15 years.

Additionally, interviews were conducted with 11 adults, divided into two groups: 6 experts in the fields of robotics, ASD, or children therapy, and 5 non-experts. The expert group consisted of psychologists, occupational therapists, and specialists in applied robotics, while the non-expert group was composed of individuals with no professional training in these areas.

The identity of the children who completed the questionnaire remained anonymous, as no identifying information was requested. The questionnaire was completed in the presence of the children’s parents to provide assistance if needed. Participants were recruited via a link shared through various channels, including parent associations and personal contacts, and completed the questionnaire using Google Forms. The participant recruitment process aimed to ensure diversity in the sample of autistic and non-autistic children.

In this study, both autistic and neurotypical children were included as a reference group, to determine which design preferences are specific to ASD as opposed to those that may be common across childhood.

Moreover, we performed an exploratory analysis by gender, although the literature on gender differences in robot preferences is still at an early stage of development. For example, recent studies have demonstrated that autistic girls exhibit fewer restricted and repetitive behaviors than autistic boys, which would suggest differences in interacting with socially interactive robots, as Robins and Dautenhahn (2014), and Kumazaki et al., (2020) have mentioned previously.

Additionally, studies in social robotics have revealed gender-related inconsistencies in expectations and perceptions towards robots, contingent upon the user profile (expert and non-expert), and biases regarding the gender of the robot even outside the context of human-like or humanoid designs, as Eyssel and Hegel (2012) have indicated in their studies. While our gender-based analysis is preliminary, examining these possible differences provides opportunities to develop more nuanced and sensitive design guidelines relevant to individual differences.

### 2.2 Instruments/Material

Quantitative and qualitative data were collected using two main instruments: an online questionnaire for children and semi-structured interviews for adults.

#### 2.2.1 Questionnaire

A questionnaire was designed to obtain information on the preferences and attitudes of both autistic children and adolescents and neurotypical children and adolescents regarding the physical appearance and personality of social robots. Access to the questionnaire was through Google Forms and consisted of several sections:

1. Introduction and Objectives: The purpose and structure of the following sections were explained, along with the estimated duration of the questionnaire.
2. Demographic Questions: Participants were asked about their gender, age, and ASD diagnosis, as well as the degree of involvement (in the case of autistic participants).
3. Robotic Preferences: This section included a set of closed-ended and multiple-choice questions (e.g., size, shape, movement, color, voice, and the presence of certain characteristics).
4. Comprehension Check: This section assessed whether the participant had understood the questionnaire and wanted to provide any advice for improving the questionnaire.
5. Drawing: This section was optional, as children were encouraged to draw their ideal robot, but those who had not yet learned to draw or did not find the activity appealing could skip it.

Responses were anonymous, and participants completed the questionnaire with the help of an adult (usually parents or primary caregivers), according to best practices for surveys involving children.

#### 2.2.2 Interviews

Semi-structured interviews lasted approximately 30–45 min and were recorded for transcription and analysis. Most interviews were conducted online, with the exception of one in-person interview with a non-expert participant.

Five non-experts and six experts were selected to participate in the interviews, each lasting 30–35 min. The selection criterion for non-experts was that they were relatives of autistic persons, while experts were professionals in the fields of social robotics or ASD, such as neuropediatricians, psychologists, and therapists.

The interviews focused on several key areas, which are included in the interview script:

1. Participants’ experiences and perceptions of technology and robots in relation to ASD.
2. The Impact of Robots on the Social and Communication Skills of Autistic children.
3. Ethical Considerations in the Design of Robots for Autistic children.
4. The challenges in designing effective ASD robots and the future expectations for such technologies.

Table 2 presents the labels, descriptions, and classification of the non-expert and expert participants who were interviewed.

**TABLE 2:** Labels, description and classification of non-experts and experts in ASD interviewed.

Labels	Description	Non-experts	Experts
Experience	Evaluates the experience someone has with the needs and preferences of autistic individuals in relation to the use of technology, specifically robots.	What experience do you have with technology and robots in relation to ASD?	Do you have any experience with the needs and preferences of autistic people in relation to technology and, more specifically, robots?
		Could you share any previous experiences where you have interacted with technology or robots designed for autistic people?	
		Do you know of any robots or technologies that help autistic people?	
Benefits	Explores how research on robots focused on emotional skills can contribute to the wellbeing and daily life quality of autistic individuals.	What do you think are the most important needs of autistic people in relation to technology and robots?	How can robot research on emotional skills benefit autistic people in their daily lives?
		What impact do you think technologies and robots could have on the lives of autistic people from your point of view?	How do you think interaction with robots could influence the social and communication skills of autistic people?
Ethic	Addresses the ethical considerations that should be taken into account when designing robots intended to interact with autistic individuals, ensuring their wellbeing and safety.		What ethical considerations should be taken into account when designing robots to interact with autistic people?
Challenges	Identifies the challenges and limitations developers face when creating robots specifically designed for autistic individuals.		What are the main challenges or limitations that developers face when designing robots for autistic people?
Differences	Considers how individual differences, such as autonomy and personal characteristics, should be taken into account when designing safe and effective technologies and social robots for autistic individuals.	What are your expectations for how technology for autistic people will evolve in the future?	What personal differences, autonomy, etc., should be taken into account to design safe and effective technologies and social robots for ASD?
Relationships	Examine how interpersonal relationships can influence the effectiveness of robot interventions for autistic individuals, considering differences such as gender or degree of ASD.	Do you think the environment of use, such as home, school, or clinical settings, affects the preferences and needs of autistic people regarding robots?	How do you think interpersonal relationships can influence the effectiveness of robot interventions for autistic people, considering possible differences, such as gender, degree of ASD, etc.,?
		Should robots designed to help autistic people consider differences such as gender, degree of ASD, and how should they be designed? What is your opinion?	
		Are the individual personality and behavior of autistic people being considered when making robot interventions for them?	
Motivations	Investigates the reasons why autistic individuals choose to participate in interventions, as well as the factors that may lead them to abandon these interventions.	What are the biggest barriers to accessing effective and safe technologies and robots for ASD?	What are the biggest barriers to accessing effective and safe technologies and robots for ASD?
		Why do you think some autistic people may want to participate in interventions? And why might they decide not to continue?	What do you think are the reasons that drive autistic people to participate in interventions? And to abandon them?
Timing	Determines the most appropriate time in an autistic individual’s life to begin interventions, especially when using specific technologies or robots.	At what age do you think we should start using therapy to help autistic people? And robots or technologies?	At what age or at what point in life do you think the intervention process for autistic people should begin? And, in the case of using specific technologies or robots for ASD?
Ideal	Describes the characteristics that the “ideal robot” should have at an institution to improve the effectiveness of robot interventions for autistic individuals.	What would the perfect robot to help autistic people be like in your opinion?	Simply put, what would be the “ideal robot” at your institution to improve the effectiveness of robot interventions for autistic individuals?

### 2.3 Procedure

The study followed a mixed-method approach. Participants first completed the online questionnaire, administered individually with the support of an adult when needed. The questionnaire was accessible through Google Forms and could be completed at home or in familiar environments.

Subsequently, semi-structured interviews were conducted with both experts and non-experts. These lasted approximately 30–45 min and were audio-recorded for transcription and subsequent analysis. Most interviews were conducted online, with the exception of one face-to-face session with a non-expert participant arranged for logistical reasons.

The co-occurrence analysis was carried out at the interview level. Each interview (with experts and non-experts) was first labeled individually, followed by the identification of co-occurrence patterns across all coded interviews. This process enabled us to identify which topics tended to emerge together across participants, rather than within individual statements.

All procedures adhered to ethical standards for research involving children and vulnerable populations. The study was conducted in accordance with the Declaration of Helsinki. Informed consent was obtained from parents/guardians for child participants and directly from adult interviewees. This study received approval from the Ethics Committee of the Miguel Hernández University of Elche (code AUT.IB.EFJ.240202).

### 2.4 Data analysis

The authors of this study come from the field of bioengineering, an interdisciplinary area that integrates biomedical engineering, robotics, social sciences, and user-centered approaches. Our training combines technical knowledge in the design and development of robotic systems with social science perspectives applied to health and inclusion. None of the authors identifies as autistic, although we have accumulated professional experience in the development of assistive technologies and in human-robot interaction research, particularly focused on neurodiverse populations. This position as primarily neurotypical researchers with a strong technical background has allowed us to analyze the quantitative data with engineering rigor, but it has also required us to be especially reflective during the qualitative thematic and co-occurrence analysis. We are aware that our interdisciplinary perspective may have influenced the interpretation of the preferences expressed by the children and the prioritization of certain ethical and design issues that emerged from the interviews. To mitigate potential biases, we conducted an iterative coding process and maintained an open attitude toward the voices of the participants.

Our analysis combined quantitative and qualitative approaches to provide a comprehensive understanding of participants’ perceptions of social robots.

Quantitative data from the questionnaire were analyzed using descriptive statistics (frequencies, percentages) and comparative inferential statistics (e.g., chi-square tests for associations between preferences and group or gender variables).

Qualitative data from open-ended questionnaire responses and interview transcripts were thematically analyzed using ATLAS.ti (version 9) which is a software to facilitate the identification of recurring patterns in preferences, perceptions, benefits, and challenges. Co-occurrence analysis was conducted to explore patterns of thematic interconnection across the 11 interviews. The analysis was performed at the interview level (rather than quotation level) to identify which themes tended to emerge together across participants, as recommended for exploratory stakeholder perspectives in mixed-methods designs.

All codes from the final codebook (developed inductively and deductively from the interview guide) were initially included in the “Code Co-occurrence Table”. Rows and columns were populated with the 9 main thematic codes (see Table 3) The default co-occurrence operator was used (overlapping or proximity within the same document), and cell values represent the raw number of interviews in which two codes co-occurred at least once.

**TABLE 3:** Co-occurrence analysis of interview codes.

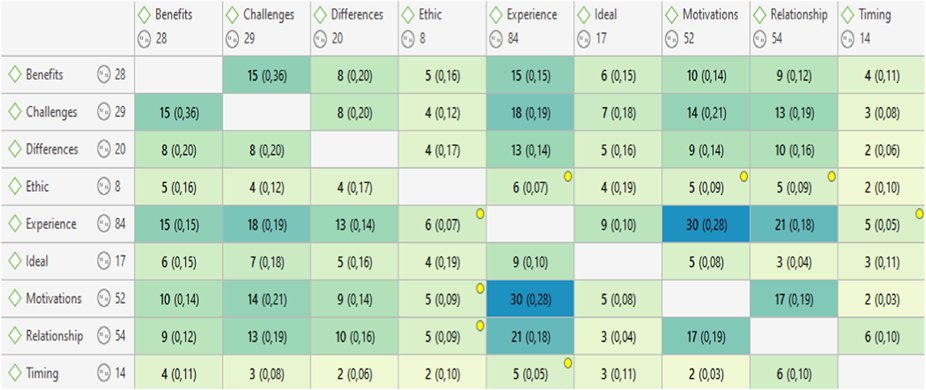

To enhance interpretability, cells were color-coded by frequency intensity (darker shades indicating higher co-occurrence counts), with a minimum threshold of 5 co-occurrences applied for visual highlighting of stronger patterns (this threshold was set *a priori* to focus on recurrent associations while avoiding noise from rare overlaps). Any statistical significance testing was applied, as the analysis was exploratory and the sample size (n = 11 interviews) was small.

The analysis and matrix generation were conducted by a single experienced qualitative researcher, who has advanced training in ATLAS.ti and thematic analysis. The resulting matrix (Table 3) highlights selected high-frequency co-occurrences (e.g., 30 instances between “Motivation” and “Experience”, 21 between “Relationship” and “Experience”) that informed the synthesis of stakeholder perspectives on robot design and ethical considerations.

## 3 Results

The results are divided into two sections: questionnaire analysis and qualitative analysis.

### 3.1 Quantitative results

Table 4 shows that most of the participants have seen a robot at some point, as the percentage of affirmative responses is quite high among neurotypical (NT) (84.62%) and autistic children (94.74%). Regarding the size of the robot, both genders of neurotypical children mentioned that the robots were small, with a relatively small difference in their percentages, unlike the group of ASD boys, of whom 63.16% indicated that the robots were large. In terms of the shape of the robot, in general all groups perceived robots as having a human form, although the most notable finding is the group of ASD boys with 84.21%.

**TABLE 4:** Participants’ preferences and perceptions of robots according to group (NT and ASD).

Questions	NT boys (%)	NT girls (%)	ASD boys (%)	ASD girls (%)
Have you ever seen any robot?	84.62	77.78	94.74	50.0
Was it big or small?	38.46 big 61.54 small	33.33 big 66.67 small	63.16 big 31.58 small	1.0 big 100.00 small
Was it shaped like a person or an animal?	53.85 person 23.08 animal	55.56 person 33.33 animal	84.21 person 5.26 animal	50.00 person 50.00 animal
Was it one color or more?	69.23 one color 30.77 more colors	55.56 one color 44.44 more colors	73.68 one color 26.32 more colors	50.00 one color 50.00 more colors
What color was it?	46.15 white 15.38 grey	11.11 grey 11.11 Brown	68.42 white 10.53 black and white	50.00 black and white 50.00 grey
Did it make any sound?	83.33 yes 16.67 No	85.71 yes 14.29 No	89.47 yes 10.53 No	100.00 yes
Did it make any particular movement?	83.33 yes 16.67 No	75.00 yes 25.00 No	84.21 yes 15.79 No	50.00 yes 50.00 No

Regarding color, responses generally indicated a preference for robots of a single color, but in the group of neurotypical girls the difference in preference was only 11.12%, with white and gray being the most mentioned.

As for the sound and movements of the robot, these are characteristics that all groups of participants perceived at fairly high rates, reaching 89.47% in the group of autistic children with respect to sound, and 83.33% in the group of neurotypical children with respect to movement.

To make a comparison between previously known aspects of robots and current preferences, it is necessary to analyze the influence of perceptions about robots on their choices.

**FIGURE 1:**
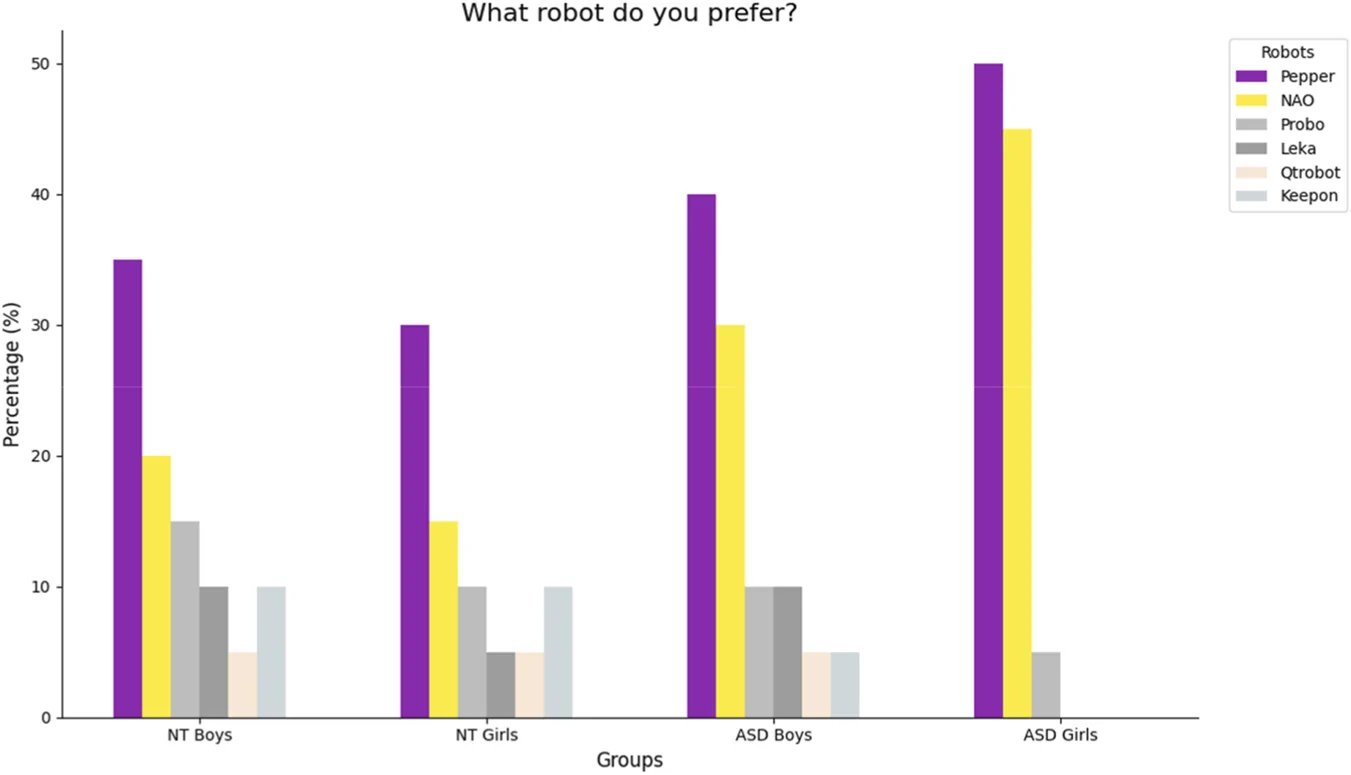
Children’s preferences for specific social robot models (Pepper, NAO, Probo, Leka, Qtrobot, Keepon) by group and gender. Percentages represent the proportion of participants selecting each robot as their preferred option (NT boys n = 13, NT girls n = 9, ASD boys n = 18, ASD girls n = 3). Note: Due to the very small number of girls with ASD (n = 3), results for this subgroup should be interpreted with caution.

To visualize how the preferences of the different groups are distributed by the type of robot (Pepper, NAO, Probo, Leka, Qtrobot, Keepon) in Figure 1. It is noteworthy that the robot Pepper is the one that receives the most votes, especially from the groups of ASD Boys, NT Boys and NT Girls, followed by NAO in all groups. The preference is more marked in the group of neurotypical children, for whom Pepper and NAO received the highest percentages, with an obvious difference compared to Leka and Probo. However, in the group of ASD and neurotypical girls there is a greater distribution in their preferences compared to the rest of the robots. Pepper was the most voted robot by autistic children with a difference of more than 20% over NAO and Leka.

Regarding the Table 5, features are compared to the variable “What robot do you prefer”. Although characteristics such as color and movement are relevant and should be considered in preferences, there are other aspects such as size, shape and sound that do not seem to influence the choices made by the groups of participants. Significant p-values are observed in some questions, for example, the question “Was it one color or more?” shows a Chi2 of 15.1844 and a p-value of 0.0189, which is below the typical significance threshold of 0.05, indicating that there is a significant relationship between the responses to this question. This suggests that the number of colors in the robot is related to other factors, such as the perception or characteristics of the robot. On the other hand, the question “Have you ever seen one?” has a p-value of 0.0933, which is above the 0.05 threshold, suggesting that there is not enough evidence to conclude that there is a statistically significant relationship between whether people have seen a robot and other variables in the study.

**TABLE 5:** Feature, chi2, p-value and degrees of freedom in relation to the question “What robot do you prefer?”.

Feature	Chi2	p-value	Degrees of freedom
Have you ever seen any robot?	10.8459	0.0933	6
Was it big or small?	8.1042	0.2306	6
Was it shaped like a person or an animal?	14.5745	0.2655	12
Was it one color or more?	15.1844	0.0189	6
What color was it?	108.3207	0.3156	102
Did it make a sound?	14.7555	0.2550	12
Did it make any particular movement?	26.0633	0.0105	12

Similarly, the question “Was it shaped like a person or an animal?” shows a Chi2 value of 14.5745 with a p-value of 0.2655, indicating no significant relationship, as the p-value is much higher than 0.05. This suggests that the shape of the robot (person or animal-like) does not appear to be strongly linked with other aspects in the dataset. Conversely, questions like “Did it make a particular movement?” with a Chi2 of 26.0633 and a p-value of 0.0105, indicate a significant relationship between the type of movement a robot made and other variables.

**FIGURE 2:**
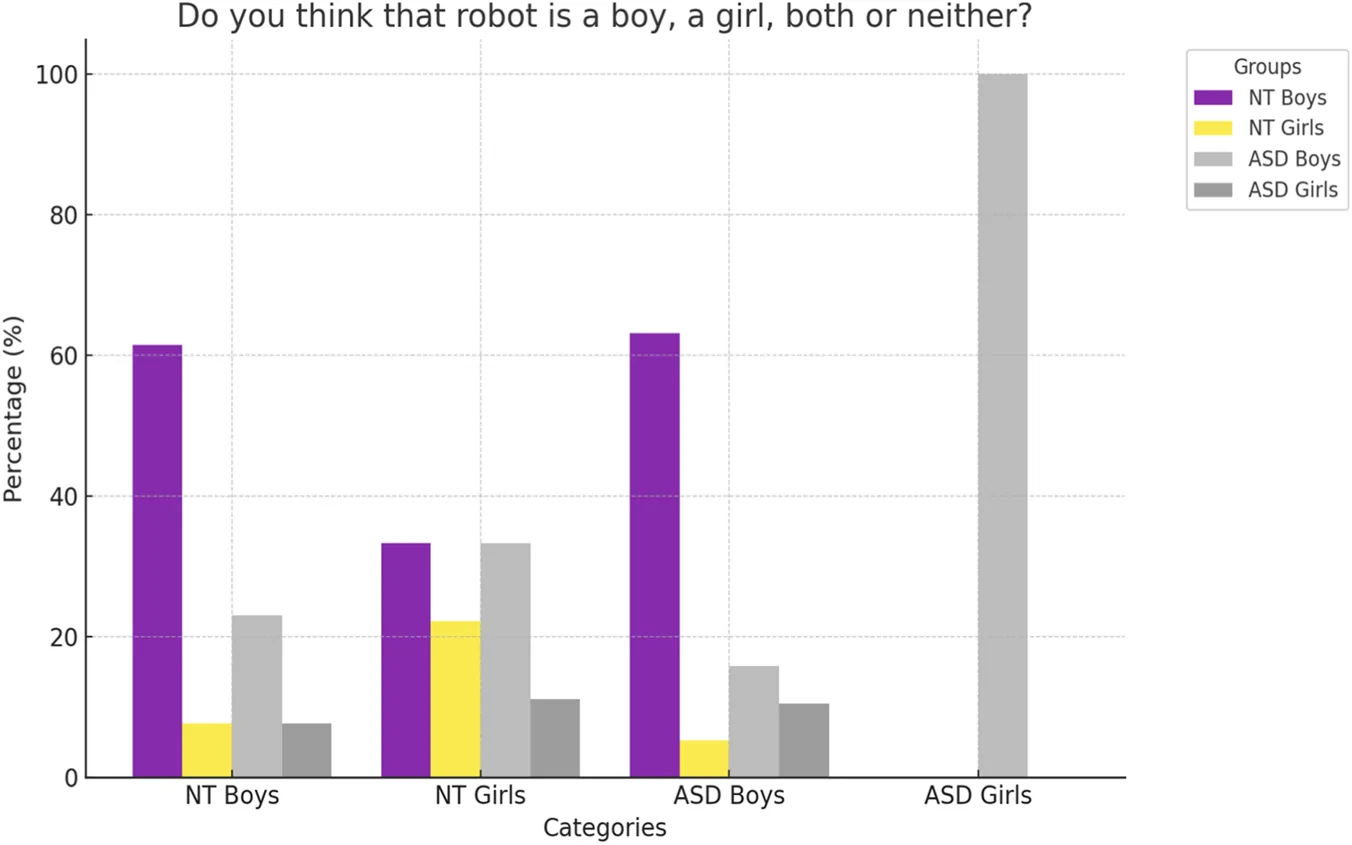
Preferences related to the sex of the social robot. Percentages by group and gender. Sample sizes as in Figure 1.

On the other hand, when analyzing the relationship between the perception and preferences of different groups towards robots, the participants’ responses are not due to chance, but their perceptions about the sex of robots in relation to the voice, are associated with their preferences about how they would like them to be, for example, boy, girl, both, or neither of the possible sexes. These data are presented in Figure 2. Moreover, the value of Chi2 = 23.36 and a value of p = 0.0054 with respect to the question “Would you like that the robot to be a boy, a girl, both or neither?” shows statistical significance.

**FIGURE 3:**
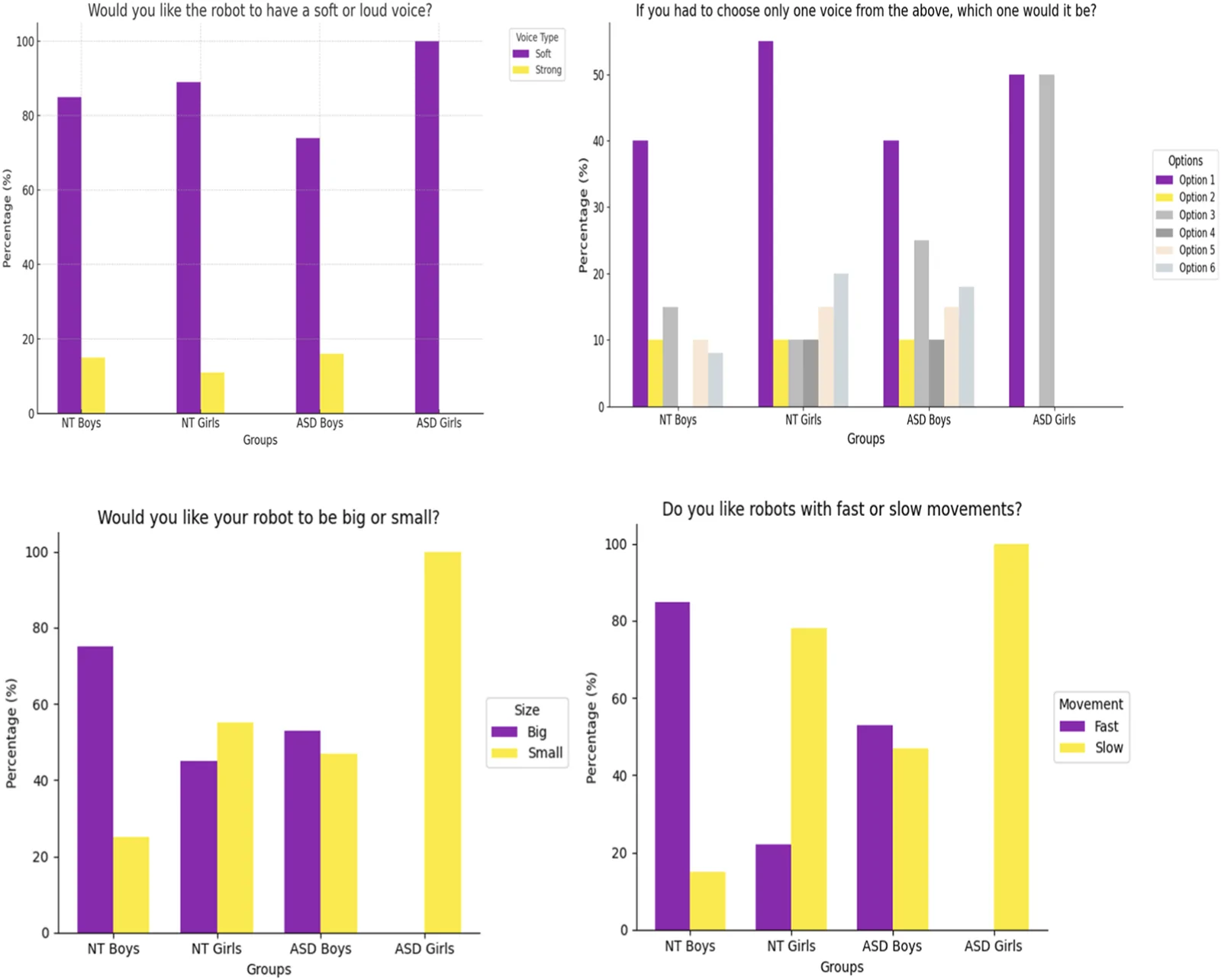
Preferences for robot voice characteristics (soft vs. loud volume and voice type) across groups and gender (up). Panel left: Preference for soft or loud voice (percentage of participants). Panel right: Preferred voice type (6 options ranging from natural human male to robotic female voices). Note: Near-unanimous preference for soft voices was observed across all groups. Preferred robot size (big/small) and movement speed (fast/slow) by group and gender (down). Error bars represent 95% confidence intervals. Sample sizes: as in Figure 1.

There is practically unanimity regarding the preference for soft or loud voices, without distinction of sex (see Figure 3, panels of down figure). The same applies to the choice of the type of voice they prefer, in the case of:

Option 1: human and natural male voice, with a soft texture, a clear and vibrant tone, subdued.
Option 2: less natural male voice, with a slightly harsh tone that could indicate that the voice is somewhat forced or unpolished, as if it were somewhat tense, not completely soft or fluid, a little robotic.
Option 3: less natural male voice very robotic, strident, rough, remains bright, but with a touch of hardness in the texture. The harsh sound could indicate that it is digitally processed, suggesting a “strength” quality in the voice.

Options 4, 5, and 6 are similar to the previous options, however, the voices were female.

The results shown in the lower panels of Figure 3 how different groups of participants chose their preferred characteristics regarding the size, movement, and capabilities of the robots. The NT Boys group shows a clear preference for large robots (76.92%) as do ASD Boys but with a smaller difference, in the NT Girls group there is a more balanced distribution between large and small robots. In terms of speed, there is a marked difference by sex, i.e., both groups of boys (NT and ASD) prefer fast robots, while groups of girls are inclined towards slow robots with a high percentage (77.78%).

**FIGURE 4:**
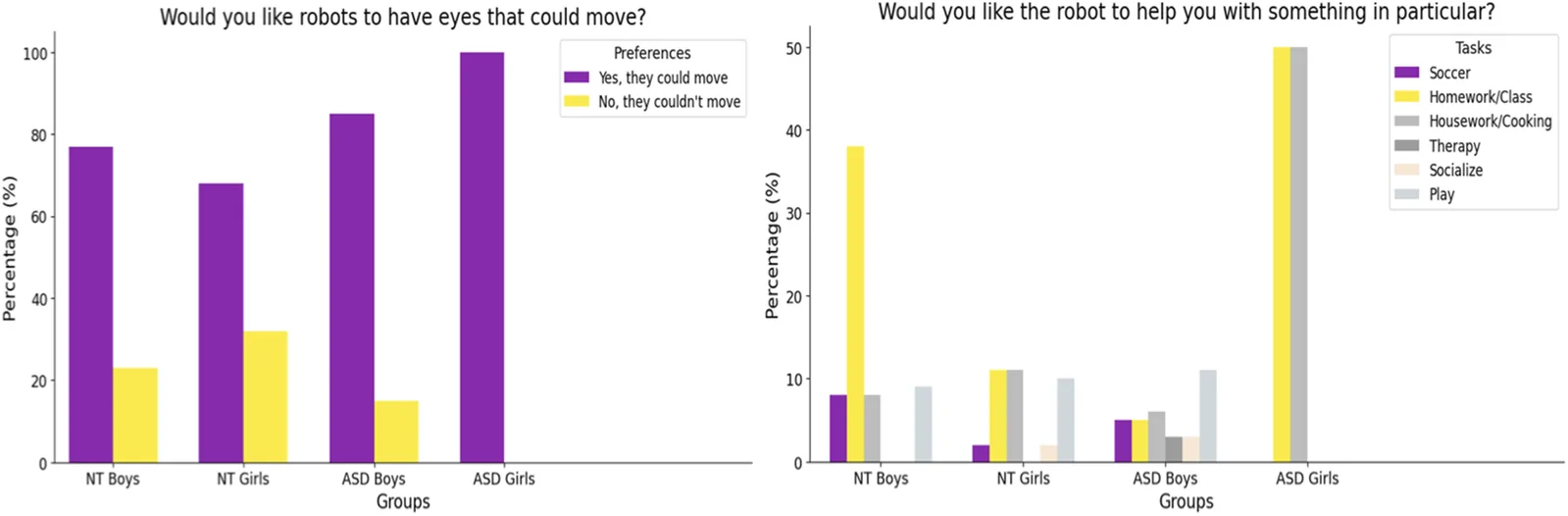
Panel left: preference for eye movement capability in social robots by group and gender. Bars represent the percentage of participants who preferred robots with moving eyes vs. static eyes. Sample sizes: as in Figure 1. Note: Strong consensus (>90% in all groups) favoring robots with moving eyes. Panel right: Preferred types of assistance or activities provided by social robots, categorized by group and gender. Response options included academic help (homework/classes), sports, playful/social activities, and others. Percentages reflect participant selections. Sample sizes: NT boys n = 13, NT girls n = 9, ASD boys n = 18, ASD girls n = 3. Note: Neurotypical children showed greater preference for academic and sports-related help, while autistic children emphasized playful and social interaction.

On the other hand, there is practically unanimity regarding the preference for eyes movement, without distinction of sex, as shown in Figure 4. Moreover, the right panel shows that NT Boys and NT Girls groups show more preference for activities such as homework/classes and sports. In contrast, there are both groups of autistic people, boys and girls, who prefer playful activities that involve socializing.

**FIGURE 5:**
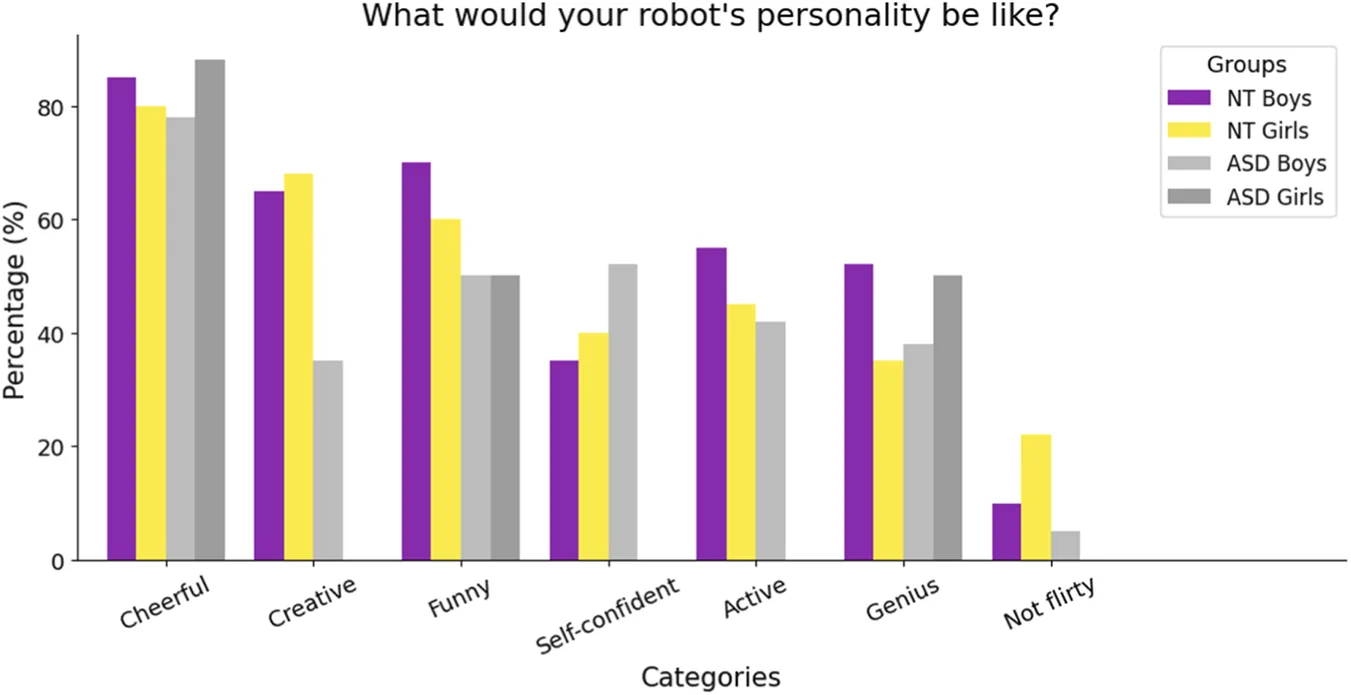
Preferred personality traits for social robots by group and gender. Participants rated traits including cheerful, creative, fun, self-confident, active, genius, and others (multiple selection possible). Bars show the percentage of participants endorsing each trait. Sample sizes: as in Figure 1. Note: Neurotypical children more frequently endorsed cheerful, creative, and fun traits, whereas autistic children showed greater variability, with some emphasis on self-confidence and creativity (particularly among ASD girls). Results for ASD girls are exploratory due to small subsample size (n = 3).

Finally, in Figure 5, the differences between the groups reflect different perceptions of personality that are influenced by gender and diagnosis. Neurotypical children’s responses indicate that they are more involved in qualities such as being cheerful, creative, and fun. On the other hand, autistic children seem to show a more dispersed distribution, with some characteristics standing out more than others, such as being more self-confident, but less active or fun. Group of ASD girls show very different traits in some attributes such as creativity and genius. In addition, 100% of participants consider that robots can be of help to autistic people, which is a very positive predisposition on the part of users.

### 3.2 Qualitative results

The results of the qualitative analysis are divided into the following subsections: Experience and motivation in interacting with robots; Experience and relationships in interacting with robots; and Experience and challenges in interacting with robots.

#### 3.2.1 Experience and motivation in interacting with robots

From the information obtained through qualitative analysis, it is observed that in some accounts, the use of robots in therapeutic interventions for autistic children can have a significant impact on the development of social skills, increased social participation, and improved communication. This is evident in the analyzed co-occurrences of the tags “Experience” and “Motivation,” “Relationship” and “Experience,” as well as “Challenges” and “Experience.” For example, some testimonies mention that children’s motivation increases when interaction with robots aligns with the family’s personal interests, influenced by social imagery, specifically as seen in some mass sports, such as football:

[…] *no, because in fact many families would surely find out about this, just because the children, let's see, the extracurricular activities of today are like if there were robotics in a school, I tell them to go to robotics because it is a sure success for an autistic person, right? Not football, not things of that kind. So just for that, because it is an interest that they have for sure that would take the families there, and we do not know it.* (Expert woman, n1).

In this case, the experience of families is connected with the school activities available for autistic children and the motivation of children through the specific interest in robotics, despite the lack of information in health or educational contexts, as can also be observed in:

[…] *no, nothing, no, in terms of technology, nothing. In fact,… We believe that at the level of technology with autism there is very little information and very few things, apart from visual agendas and programs for communication, which are also very complicated… They love it* (Expert woman, n1).

Although there is a lack of tools adapted to autistic children that facilitate their learning and communication, when this happens, children’s motivation increases. This is enhanced if technology offers an attractive way to interact and encourage their participation, considering their interests and motivations, which are often different from those of neurotypical children. Moreover, some discourses comment aspects related to the concept of how the robotic can help autistic children, for example, the motivation of autistic children is also linked to the experiences that make it possible for them to interact more autonomously and participate in daily activities independently, a key point in their vital development.

[…] *that they can teach them skills, for example, I think safety on the street is an important thing, or when they grow up, for example, another thing that I think would make the family go, I am thinking about our teenager all the time, for example, this family is doomed, in quotation marks, because she can never do anything alone. She can do absolutely no activity of daily life alone. So, that, teaching, that they can do a more or less normalized activity, because in the end they are very deprived of being able to do things. So, that you motivate the child, you know? Because you take a autistic child to the zoo and what a typically developing child loves. You give the autistic child a mobile phone or if he wants, normally, from the zoo, with robots that would surely be a motivation (Expert woman, n2).*

#### 3.2.2 Experience and relationships in interacting with robots

Family motivation and optimism towards the use of robots and other technologies can have a positive impact on children’s engagement and emotional learning. Regarding interpersonal relationships, particularly in the case of children, the relationship with their parents can influence the perceived usefulness of these robot-supported activities, so it is important that families remain involved and committed to support the child’s participation. In general, experts reported that the use of social robots may have positive effects on family relationships.

[…] *When family and closest relationships are involved in an intervention… there will always be greater adherence of that person and greater… It's a placebo thing in the end, isn't it? If your environment is excited about an intervention, in the end what can be achieved is brutal, isn't it? And maybe your roof is here, but well, you’re going to get to the ceiling and even people with that optimism and that acceptance of the intervention, because in the end you improve the circle, right? And you improve the relationships of… that you have with your parents, with your people (Expert woman, n3).*

The interpersonal connections that professionals in the field of ASD can maintain with parents of children with different degrees of ASD and their comorbidities, also represent new forms of support for families, through sharing information and experiences that encourage them to explore new technologies and tools, such as the use of social robots:

[…] *Here, to give you an idea, we have all degrees of ASD, but up to 14 years old. Well, the oldest is 15 right now, so it’s more early care. So, yes, we would like it, because until the children mother, told me about it, we, look, we are here in the center of Madrid, we had no idea about any of this. Then I started to get into the internet, I have seen videos especially of Pepper, I have also seen some of the boy, with the robot and others, but we had no idea about any of this (Expert woman, n4)*.

Additionally, the experience of therapists and families can influence the approach of therapies, as it is still a limited tool in terms of certain aspects of social and emotional development, considering the robot as a complement to human work in interventions.

[…] *it would be well planned as long as it is in that is, not exclusively the work with this, but that the robot is like a preliminary for a later work, that is, that it is not limited only to the work with the robot, because in the end it is that, I think there is a part… emotional and spontaneity and such, that a robot will never have it, of course, and that they also need, you need to work, of course (Expert woman, n1)*.

#### 3.2.3 Experience and challenges in interacting with robots

Despite the experience of families and professionals in the fields of ASD and robotics engineering, a set of ethical challenges arise. The possibility of negative effects arising from interaction with robots is raised, such as children perceiving a robot as a real friend and developing emotional dependence. Addressing this problem, the concerns that arise when designing robots to interact with children must be taken into account.

[…] *If a child or a teenager with ASD relates to that robot as if it were their friend, which I have seen in a movie, the typical child who becomes obsessed with the robot and then considers it their partner, I don't know what, that’s like a little… But well, I want to tell you, this girl we were talking about before, who talks to Coco, who is a crocodile from a game singer, who is not a real character either. Then… (Expert, woman, 2)*.

In the same way, other alternatives for the use of robots must be offered, such as being used to produce an improvement in the planning of activities and tasks, and reinforcement of the skills acquired. To do this, it is proposed to follow a sequence of steps into which the task has been divided, adjusting to the different levels of needs and responses of each child, and providing reinforcement as a reward that guarantees motivation and follow-up.

[…] *It would have to be like with… a lot of sequencing in steps that helps a lot in the planning of what they have to do and a lot of reinforcement in between that, a lot of reinforcement in between the steps. And for example, if you are going to accompany him to go out on the street, once again the planning of all the steps, of all the situations that may happen, alarm signals, all that with a lot of reinforcers (Expert woman, 1)*.

Another of the great challenges on which a large part of the interviewees, both professionals and non-experts, agree is the barrier to access to useful technologies. Although there are powerful technological tools such as visual agendas or programs to improve their communication, they are complex to handle for both adults and children, which is a great limitation for technology developers, especially robots.

[…] *No, nothing, no, technology-wise, nothing. In fact, … We believe that at the level of technology with ASD there is very little information and very few things, apart from visual agendas and programs for communication, which are also very complicated, very difficult and such, apart from that nothing (Expert woman, n1)*.

### 3.3 Synthesis of findings

When combined, the quantitative and qualitative findings provide a comprehensive view of children’s preferences and adults’ perceptions of the social robots for ASD interventions.

Overall, there were similarities and differences across the groups. Despite the clear role of diagnostic group and gender, children generally preferred robots with mobility, sound, and eye movements. These features were perceived as essential for making robots engaging and enhancing aspects of social interaction. More importantly, all participants (NT and ASD) seemed to believe that robots could be useful for autistic children, demonstrating a positive predisposition toward the therapeutic utility of robots.

The differences across groups were clear. Although children generally had similar perceptions, differences were apparent. ASD boys generally preferred larger and faster prototypes, whereas NT girls preferred smaller and slower robots. NT children endorsed the robots as having utility for academics or sports, while ASD children preferred to emphasize play and social dimensions. Preferences on robot personality also differed: NT participants mentioned traits such as cheerfulness, creativity, and fun, while ASD children made more varied and personal choices, sometimes placing higher value on self-confidence or creativity than on sociability.

In addition, experts considered ethical issues, such as the danger of emotional dependency, as well as the accessibility issues, and highlighted ensuring that robots complement, not replace, human relationships. They also emphasized safeguarding children’s wellbeing with careful design. Non-expert respondents, including family members or autistic individuals focused on motivation, daily support, and how robots might provide opportunities for autonomy and participation in daily activities.

## 4 Discussion

The findings from the quantitative analysis reveal that a significant majority of participants, including both NT and ASD children, have encountered or are familiar with social robots at some point in their lives. This is the new normality in a global and increasingly technological world, as robotics has become an integral part of daily life, and childhood is no exception. Social robots, designed to interact intuitively with people, are gaining popularity in educational and home environments, facilitating learning and skill development in children from an early age (Belpaeme et al., 2018) which applies to both neurotypical and autistic children. Some of the most recognized robots for autistic children include Paro, Leka, Pepper, NAO, and Milo.

When considering the size perception of social robots, NT children generally perceive these robots as smaller, whereas ASD boys tend to see them as larger. The preference among NT children for larger robots, especially among boys, is consistent with the findings of Robins and Dautenhahn (2014), Robins et al. (2006), Robins et al. (2005) and, Konkle and Oliva (2011) who discovered that children interacted more with human-sized robots. However, the preference of ASD girls for smaller robots is consistent with the studies by Kumazaki et al. (2020) which demonstrated that autistic children tend to feel more comfortable with small robots, possibly because they find them less intimidating.

The data on the most well-known robots for autistic children indicate that 63.16% perceived robots as large, despite many widely used robots (such as Paro, Leka, NAO, and Pepper) being small or medium-sized. This discrepancy could be due to subjective size perception in autistic children, as studies suggest that autistic individuals may have enhanced luminance and contrast discrimination but differences in motion processing and facial recognition (Eyssel and Hegel, 2012). Additionally, age may also play a role, as children under 8 years old struggle to integrate visual and tactile information for size perception (Gori et al., 2008), consistent with evidence that autistic individuals integrate prior knowledge into visual perception to a lesser extent than neurotypical peers (Ropar and Mitchell, 2002), and contextual factors can further influence visual object perception (Faghihi et al., 2015). However, findings on this topic remain inconclusive, as some studies have reported no significant differences in size discrimination between autistic children and neurotypical peers (Simmons et al., 2009).

### 4.1 Interpretation of quantitative findings

This discrepancy in size perception aligns with previous research by Simmons et al. (2009), who posited that the subjective perception of larger size among autistic children could be attributed to differences in light and contrast discrimination.

Across all groups, there is a consistent preference for human-like robots, which can be explained by the principle of cognitive congruence. According to Festinger (1962) theory of cognitive dissonance, individuals seek consistency between their beliefs and perceptions, aiming to minimize cognitive incongruence. In this context, children may be more inclined to interact with robots that resemble humans, as this alignment reduces cognitive effort. Moreover, gender stereotypes, as noted by Eyssel and Hegel (2012) influence how children perceive and choose robots, reinforcing pre-existing gender norms. Martin and Ruble (2004) further argue that gender development in children leads them to internalize gender categories, which may explain the tendency to prefer robots that align with these categories.

Accordingly, the phenomenon of anthropomorphism, highlighted by Duffy, (2023) allows people to attribute gender to robots and make choices based on these perceptions. This effect could also be extrapolated to children, influencing the way they interact with technology. The principle of cognitive ease suggests that humans prefer stimuli that are easier to process mentally (Faghihi et al., 2015). This principle could explain why children are more likely to choose robots whose gender matches their pre-existing expectations, as it reduces cognitive load.

Interestingly, despite potential differences in social processing among autistic children, the findings indicate that preferences for robot gender are similar to those of neurotypical children, underscoring that underlying cognitive and social processes in this context are universal. These observations not only illuminate the nuanced ways different groups interact with and perceive technology but also emphasize the importance of designing robots that cater to these diverse perceptual needs and preferences.

In terms of color, the analysis reveals a predominance of robots perceived as single-colored; sound and movement are noted by the majority. Interestingly, however, the flashier movements and colors have an impact on choices of both NT and ASD children, which is a key feature in the design of robots designed to interact with a variety of people. Going into detail, with regard to the aspects that show a significant relationship with the preferences of the participants, the number of colors, that is, the presence of multiple colors seems to be more attractive to ASD participants and influence their choice.

In the literature there are discrepancies about the predominant color to be used in social robots: Dou et al. (2022) recommend the use of light colors for the head, neutral colors favored for their high acceptance, competence evaluation, and low discomfort, with warmth judgment akin to warm colors and competence rating similar to cold colors. However, Lin et al. (2022) advocate for choosing a robot’s color based on the emotion it represents (positive or negative). In their analysis, they mention that people prefer the color blue regardless of the facial emotion. However, they emphasize that the decision should be based on ensuring comfortable reactions for interactions with social robots. In the case of autistic children, it would be necessary to explore a more efficient response or expand the research on color preference, as sensory perception and emotional responses may differ.

Understanding these nuances could lead to more inclusive and user-friendly robotic designs, enhancing engagement and interaction quality for neurodivergent children. Moreover, color can be related to preferences regarding sound in social robots, as both neurotypical and ASD individuals indicated that they perceive robots as producing sounds and movements that influence their overall experience.

For autistic children, who may have heightened or altered sensory sensitivities, the integration of appropriate sound design could be just as important as color selection in ensuring a positive and non-disruptive interaction. Therefore, a multisensory approach that carefully considers both visual and auditory aspects could contribute to the development of more adaptive and inclusive social robots. The same applies to the specific movements of robots, which are strongly associated with their preferences. Movement can be an important factor that makes the robot being perceived as more attractive and familiar to autistic children.

In this sense, qualitative results complement quantitative ones by highlighting the expectations and concerns of stakeholders.

### 4.2 Interpretation of qualitative and stakeholder insights

According to social robots’ voices, the data analyzed in the questionnaire generally show a trend towards preferring soft voices. Despite these findings, it should not be overlooked that in the design of social robots, autistic children might have a higher sensitivity to auditory stimuli. High-pitched or loud voices can lead to rejection, which justifies their preference for softer voices (Lemaignan et al., 2024; Shamsuddin et al., 2012). This aspect is advantageous for establishing positive human-robot interaction, as soft and friendly voices are more effective in creating an emotional connection between humans and robots, regardless of age or neurological condition (Robins et al., 2005). Moreover, softer voices enhance information retention maintain engagement, and reduce anxiety (Gomes et al., 2008). Soft voice is a universal trait, consistent across diverse cultures, highlighting its importance in designing robots that can interact harmoniously with people, particularly those who might be more sensitive to sensory inputs like autistic children.

Regarding model preferences for social robots, Pepper and NAO are the most favored robots, with a general preference for soft voices, varying in naturalness and gender. Boys prefer large and fast robots, whereas girls opt for small and slow ones. There is unanimity in the preference for eye movement in robots. The preferred activities for robot assistance differ: NT children prefer academic and sports help, ASD children seek playful social activities. In terms of personality, NT children value cheerfulness, creativity, and fun, while ASD children show more varied preferences, highlighting confidence and creativity, especially among ASD girls.

A major limitation of this study is the relatively small overall sample size (N = 43 children) and, in particular, the severe imbalance in the gender distribution within the ASD group, where only three girls participated (n = 3 ASD girls vs. n = 18 ASD boys). This imbalance is consistent with the well-documented higher prevalence of ASD diagnoses in boys compared to girls (approximately 4:1 ratio in many epidemiological studies), and recruiting a more balanced sample of ASD girls proved particularly challenging within the recruitment timeframe and resources available.

Consequently, any findings related to gender differences, especially those concerning preferences expressed by ASD girls, must be regarded as purely exploratory and preliminary. The extremely low number of ASD girls severely limits the statistical power to detect true gender effects, increases the risk of Type II errors (failure to detect existing differences), and makes the results highly susceptible to sampling variability and individual idiosyncrasies. In addition, the small subsample size prevents meaningful subgroup analyses (e.g., by age within gender or by ASD severity level) and precludes robust generalization of gender-related patterns to the broader population of ASD girls.

Although the inclusion of a neurotypical comparison group and the mixed-methods design provide valuable initial insights, the gender comparisons reported here should be interpreted with caution. Statements about consistent gender differences in robot preferences (e.g., size, movement speed, personality traits) remain tentative at best and should not be taken as conclusive evidence. Replication in larger, more gender-balanced samples (ideally with substantially more ASD girls) is essential to confirm, refute, or refine these observations. Future studies should also consider stratified recruitment strategies or targeted oversampling of girls to achieve adequate statistical power for gender-specific analyses.

Furthermore, the small number of ASD girls in the sample may have inadvertently amplified the influence of any unmeasured individual differences (e.g., co-occurring conditions, intellectual ability, sensory profile, or prior exposure to robots) within this subgroup, further complicating the attribution of observed patterns to gender *per se* rather than other confounding factors.

In summary, while the present findings offer promising directions for user-centered social robot design in ASD in autism-related contexts, the gender-related results should be viewed as hypothesis-generating rather than hypothesis-confirming. Strong conclusions about gender-specific preferences would be premature without replication in adequately powered studies.

In general, these data obtained by the questionnaire in ASD participants are consistent with well-known robots, such as the humanoid and the predominantly white color, which correlates with the description of robots like NAO and Pepper. The same applies to sound and movement, as both robots are designed to interact through sounds and movements (Puglisi et al., 2022).

**FIGURE 6:**
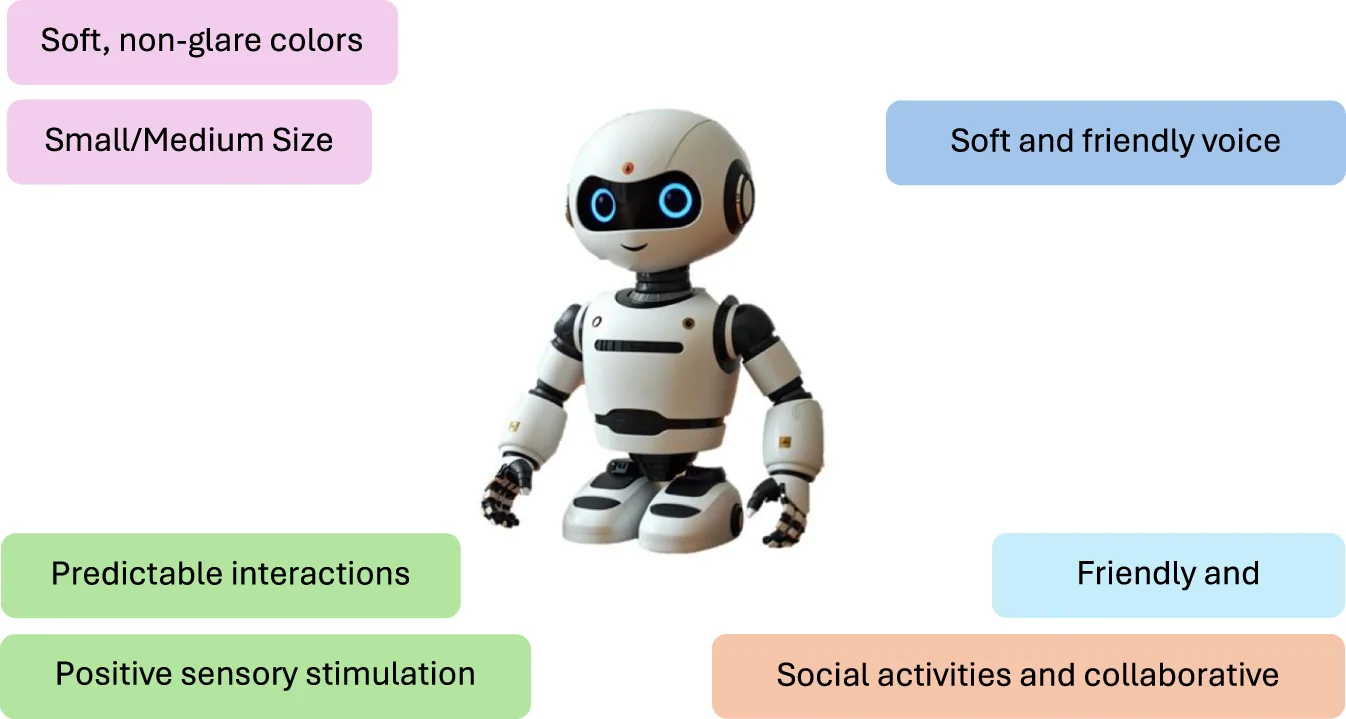
Conceptual prototype of an “ideal” social robot for autistic children, synthesized from the most frequently preferred design features reported by ASD participants (humanoid shape, moderate size, soft voice, moving eyes, multi-color elements with moderate intensity, playful interaction focus). Image generated using Fotor AI tool based on aggregated qualitative and quantitative preferences. This is an illustrative representation, not a functional design.

The results of qualitative analysis indicate that interacting with robots in the therapeutic context of autistic children could increase the impact of social and communicative development. Social robots have the capacity to align with the interest of child participants. Following the results extracted from the quantitative and qualitative analysis, the characteristics chosen by autistic children can be summarized and visualized through a robot prototype approximation in Figure 6.

Moreover, families expressed positive attitudes towards the use of social robots in support contexts, viewing them as a potentially valuable technological complement for fostering new forms of interpersonal interaction and support. For example, the codes “Motivations” and “Ideal” suggest that interviewees, both experts and non-experts, link their motivations to their ideal concepts, highlighting how aspirational and emotional aspects are closely connected. In this regard, personalization in assistive robots has been reinforced as a key factor for improving interaction during the last years. Especially following the integration of generative artificial intelligence (AI) tools. Generative AI could facilitate a future research agenda for diverse applications, which includes Human-Robot Interaction (HRI), such as Su et al. (2023) and Obrenovic et al. (2024) propose.

Additionally, current robots in multi-user environments require adaptation to produce personalized interactions, which could also transform the current research in HRI with children, particularly referring to non-neurotypical groups, such as autistic children. In these scenarios, the user’s feedback leads the robots to learn from experiences and use this knowledge to generate adapted activities to the user’s preferences. However, preferences are user-specific and may vary, so learning is required to personalize the robot’s actions to each user.

In addition, our co-occurrence analysis shows that certain ethical considerations are less frequently linked to other aspects, indicating that participants treat ethical discussions in a more isolated way. This information helps guide future robot designs by identifying which topics might be more complex or less integrated among users. Robots can obtain feedback in HRI by asking users their opinion about the activity (explicit feedback) or estimating it from the interaction (implicit feedback), such as Maroto-Gómez et al. (2024) suggest. Thus, by combining explicit and implicit user feedback produces better adaptive behavior in robots than considering them separately, leading to higher engagement and more tailored activities for each user.

### 4.3 Implications for design and future directions

The findings of this study have direct implications for inclusive design and point to future opportunities in social robotics. From the perspective of Aoudni et al. (2025) and, it is proposed to use behavioral artificial intelligence within the modern environment framework to personalize HRI. The ability of entertainment robots (enbots) to detect, understand, and react to our emotions can lead to a higher degree of engagement and camaraderie. These robots can adjust their actions, appearances, and responses based on personal desires and psychological states, creating more personalized and engaging interactions. However, these enbots still cannot fully identify human emotions. The study of Aoudni et al. (2025) proposes a method to adjust the enbots’ features based on an individual’s psychological state, using electroencephalography (EEG) technologies to digitize and interpret human emotions. The enbot settings will be automatically modified to keep the individual’s emotions within a desired range, thus increasing mutual trust and respect between human and robot.

Moreover, the results of our analysis are aligned with and extend the principles of inclusive design and the neurodiversity paradigm, which advocate for robotic systems that are flexible, modifiable, and respectful of individual neurological and gender-based differences rather than imposing normative expectations on behavior (Safavi et al., 2024; Eyam et al., 2021). By highlighting that gender differences in size and movement speed preferences were more consistent than diagnostic differences, the present study underscores the need to move beyond a solely deficit-based approach to ASD and incorporate gender-sensitive and neurodiverse design criteria from the earliest stages of robot development. This critical synthesis reveals a persistent gap in the literature: while outcome-focused studies dominate, user-preference and inclusive-design studies remain scarce, limiting the ecological validity and real-world applicability of current social robots (Scassellati et al., 2018).

According to the limitations of social robots, it is evident in the works of other researchers the technological divide (Kumm et al., 2022) and the scarcity of social robots specifically designed to meet the genuine needs and demands of autistic children and their families. This technological gap not only highlights the disparity in access to advanced technologies but also underscores the urgent need for the development of social robot solutions that are inclusive and tailored to the particularities of the autistic children.

The positive preferences towards robots in autistic children suggest a significant opportunity for improving their quality of life and social interaction, offering valuable support to both them and their families. However, a significant technological divide still exists that affects equity in access to these innovations. This divide is evident in the availability of resources, the cost of technology, and the preparedness of professionals to implement robotic solutions in therapeutic or educational settings.

Nevertheless, social robotics in therapy with children is undergoing rapid advancement, largely due to developments in artificial intelligence (AI). AI is enhancing robots with improved facial recognition, natural language understanding, and adaptability to individual needs, which can further personalize interventions for autistic children. Projects like the use of the Pepper Robot in schools have shown how these robots can facilitate teaching and socialization, which could be reinforced with emerging models of AI.

The acceleration of AI promises not only to narrow the technological divide but also to make these technologies more accessible and affordable. For instance, current research focuses on creating low-cost, open-source robots, like the NAO Robot project, which could be easier to adopt in various contexts in school, as other projects have demonstrated evidence of improving learning skills in the children population (Kennedy et al., 2015; Belpaeme et al., 2018; Jacob et al., 2025).

Thus, in the near future, robotic interactions are expected to become more inclusive, by improving wellbeing, social inclusion, and learning outcomes for autistic children and all children alike. This approach will foster an inclusive educational environment where robotics may contribute to diverse learning needs, promoting cognitive, social, and emotional development through tailored interaction and adaptive learning strategies. By providing personalized learning experiences, these interventions aim to bridge educational gaps, enhance engagement in academic tasks, and provide children with the necessary skills for future challenges, making education accessible and effective for every student, regardless of their abilities or learning styles.

All these findings emphasize previous related work, contextualizing our study within the recent literature on social robots in ASD therapy, which highlights their potential to improve social and communication skills. For example, Kumazaki et al. (2020) showed that robots such as NAO and Pepper foster joint attention, while Chung (2019) highlighted the effectiveness of social robots in engaging children in therapeutic settings. Meanwhile, Lemaignan et al. (2024) showed that Pepper supports socialization in schools, supporting the familiarity reported by children in our study. In terms of design, Robins et al. (2005); Robins et al. (2006) found that autistic children interact better with human-sized robots, although Kumazaki et al. (2020) suggested that small robots are less intimidating, consistent with the preference of ASD girls for small robots in our data. Dou et al. (2022) recommend neutral colors to improve acceptance, in line with our observation of preference for single-tone colors, and Gomes et al. (2008) highlight the importance of soft voices, supporting the unanimous preference for soft voices across all groups. Maroto-Gómez et al. (2024) and Aoudni et al. (2025) propose personalization through feedback and emotional intelligence, supporting the need for adaptive designs identified in this study. However, Kumm et al. (2022) point to a technological gap that limits access, and warn of ethical risks such as emotional dependence, reflected in our interviews (Belpaeme et al., 2018). This study addresses the paucity of comparative research between autistic children and neurotypical children, integrating quantitative and qualitative data. We propose exploring low-cost robots and generative artificial intelligence to personalize interactions and improve inclusion.

### 4.4 Limitations

This study has several important limitations that must be acknowledged when interpreting the findings and the future patterns. First, the overall sample size of children (N = 43) is relatively small, particularly when considering the need to conduct subgroup analyses by diagnosis (autistic children vs. neurotypical) and gender. More critically, the ASD group showed a severe gender imbalance, with only three ASD girls participating (n = 3) compared to 18 ASD boys. Although this distribution reflects the well-established higher prevalence of ASD diagnoses in boys (approximately 4:1 man-to-woman ratio), the low number of ASD girls restricts the statistical power and reliability of any gender-specific conclusions within the ASD group. Findings related to preferences among ASD girls (for example, robot size, movement speed, or personality traits) should therefore be regarded as exploratory. In this sense, the results are particularly susceptible to sampling variability, individual differences within the small subsample, and potential confounding factors (such as co-occurring conditions, sensory profiles, intellectual functioning, or prior technology exposure) that could not be adequately controlled or explored in our analysis.

Second, the recruitment strategy relied heavily on convenience sampling through parent associations, personal contacts, and online dissemination, which may have introduced selection bias toward families already interested in or familiar with technology and robotics. This could limit the generalizability of the findings to broader populations of autistic children and neurotypical children, particularly those from less technologically engaged or socio-economically disadvantaged backgrounds.

Third, the qualitative analysis, including thematic coding and co-occurrence matrix generation in ATLAS.ti, was performed by a single researcher with advanced training in qualitative methods. Although this ensured analytical consistency throughout the process, the absence of multiple coders and formal inter-rater reliability testing represents a limitation. This may have introduced some degree of researcher bias and reduced the confirmability of the identified themes and co-occurrence patterns. Including multiple coders in future research would help mitigate this limitation and enhance the trustworthiness of the qualitative results.

Finally, although the mixed-methods design provided rich complementary insights, the cross-sectional nature of the data prevents any inference about causality or long-term effects of specific robot design features on children’s engagement or therapeutic outcomes.

For all of these reasons, the limitations of our analysis highlight the preliminary nature of our analysis and underscore the need for replication in larger, more balanced, and more diverse samples, as well as for studies employing longitudinal designs.

## 5 Conclusion

In general, the participating children perceive social robots as familiar elements, having previously interacted with some type of robot and demonstrating a high degree of familiarity with them. This evidence suggests that human-robot interaction (HRI) and the presence of robots in everyday life the presence of robots in everyday life may be becoming increasingly common in childhood, which could shape future experiences with these technologies during adolescence. However, some challenges remain in the morphological design of social robots for interacting with autistic children. In this regard, it is worth noting that there is no conclusive evidence of a generalized distortion in size perception between autistic children and neurotypical children. Therefore, future research could focus on unraveling the differences in visual processing that influence human-robot interaction, especially in autistic children. Furthermore, visual characteristics (such as colors and movements) influence children’s preferences more significantly than sound or shape, indicating that robot design should prioritize attractive and captivating visual stimuli. Moreover, autistic children tend to prefer smaller, less intimidating robots, while neurotypical children, particularly boys, lean towards larger robots, highlighting the need for personalized design. This includes the widespread preference for humanoid robots, influenced by the principle of cognitive congruence and gender stereotypes, with a greater emphasis on color variety in the preferences of autistic children. Additionally, sound design, especially through soft, friendly voices, is noteworthy due to the auditory sensitivities characteristic of ASD. Robots like Pepper and NAO are the most highly rated in this analysis, with clear variations in preferences based on gender. Finally, the integration of artificial intelligence into robotics promises much more personalized interactions, although a significant technological gap persists, limiting equitable access to these innovations. In short, future developments should aim toward more inclusive robotics. The present findings, although preliminary, suggest that personalised and accessible designs have the potential to improve cognitive, social, and emotional development in autistic children and neurotypical peers alike.

## Data Availability

The raw data supporting the conclusions of this article will be made available by the authors, without undue reservation.

## Footnotes

1 Note on terminology: throughout this manuscript, we use identity-first language (e.g., “autistic children”) in line with the preferences documented among autistic communities and the APA Inclusive Language Guidelines. However, we note that language preference is not universal: Buijsman et al. (2023) found that 68.3% of Dutch autistic adults and 82.5% of parents of autistic children preferred person-first language (“person with autism”), highlighting that preference varies by language, culture, and individual. Following their recommendation, we advocate for sensitivity to context when applying these guidelines across linguistic communities. Moreover, regarding the terms such as “intervention” and “treatment” we consider that while standard in the HRI field, we acknowledge their limitations from a Critical Autistic Studies perspective (Elmadagli and Renoux, 2023; Spiel et al., 2019; Richardson, 2018). Since this study examines design preferences rather than administering clinical treatment, these terms should be read broadly as structured robot-assisted sessions.
